# VWD domain stabilization by autocatalytic Asp‐Pro cleavage

**DOI:** 10.1002/pro.4929

**Published:** 2024-02-21

**Authors:** Noa Yeshaya, Prashant Kumar Gupta, Orly Dym, David Morgenstern, Dan Thomas Major, Deborah Fass

**Affiliations:** ^1^ Department of Chemical and Structural Biology Weizmann Institute of Science Rehovot Israel; ^2^ Department of Chemistry and Institute for Nanotechnology & Advanced Materials Bar‐Ilan University Ramat‐Gan Israel; ^3^ Department of Life Sciences Core Facilities Weizmann Institute of Science Rehovot Israel; ^4^ De Botton Institute for Protein Profiling, Nancy and Stephen Grand Israel National Center for Personalized Medicine Weizmann Institute of Science Rehovot Israel

**Keywords:** Asp‐Pro bond, *cis* peptide, domain stability, mucus, multi‐domain protein, proteolysis

## Abstract

Domains known as von Willebrand factor type D (VWD) are found in extracellular and cell‐surface proteins including von Willebrand factor, mucins, and various signaling molecules and receptors. Many VWD domains have a glycine‐aspartate‐proline‐histidine (GDPH) amino‐acid sequence motif, which is hydrolytically cleaved post‐translationally between the aspartate (Asp) and proline (Pro). The Fc IgG binding protein (FCGBP), found in intestinal mucus secretions and other extracellular environments, contains 13 VWD domains, 11 of which have a GDPH cleavage site. In this study, we investigated the structural and biophysical consequences of Asp‐Pro peptide cleavage in a representative FCGBP VWD domain. We found that endogenous Asp‐Pro cleavage increases the resistance of the domain to exogenous proteolytic degradation. Tertiary structural interactions made by the newly generated chain termini, as revealed by a crystal structure of an FCGBP segment containing the VWD domain, may explain this observation. Notably, the Gly‐Asp peptide bond, upstream of the cleavage site, assumed the *cis* configuration in the structure. In addition to these local features of the cleavage site, a global organizational difference was seen when comparing the FCGBP segment structure with the numerous other structures containing the same set of domains. Together, these data illuminate the outcome of GDPH cleavage and demonstrate the plasticity of proteins with VWD domains, which may contribute to their evolution for function in a dynamic extracellular environment.

## 1 INTRODUCTION

**FIGURE 1:**
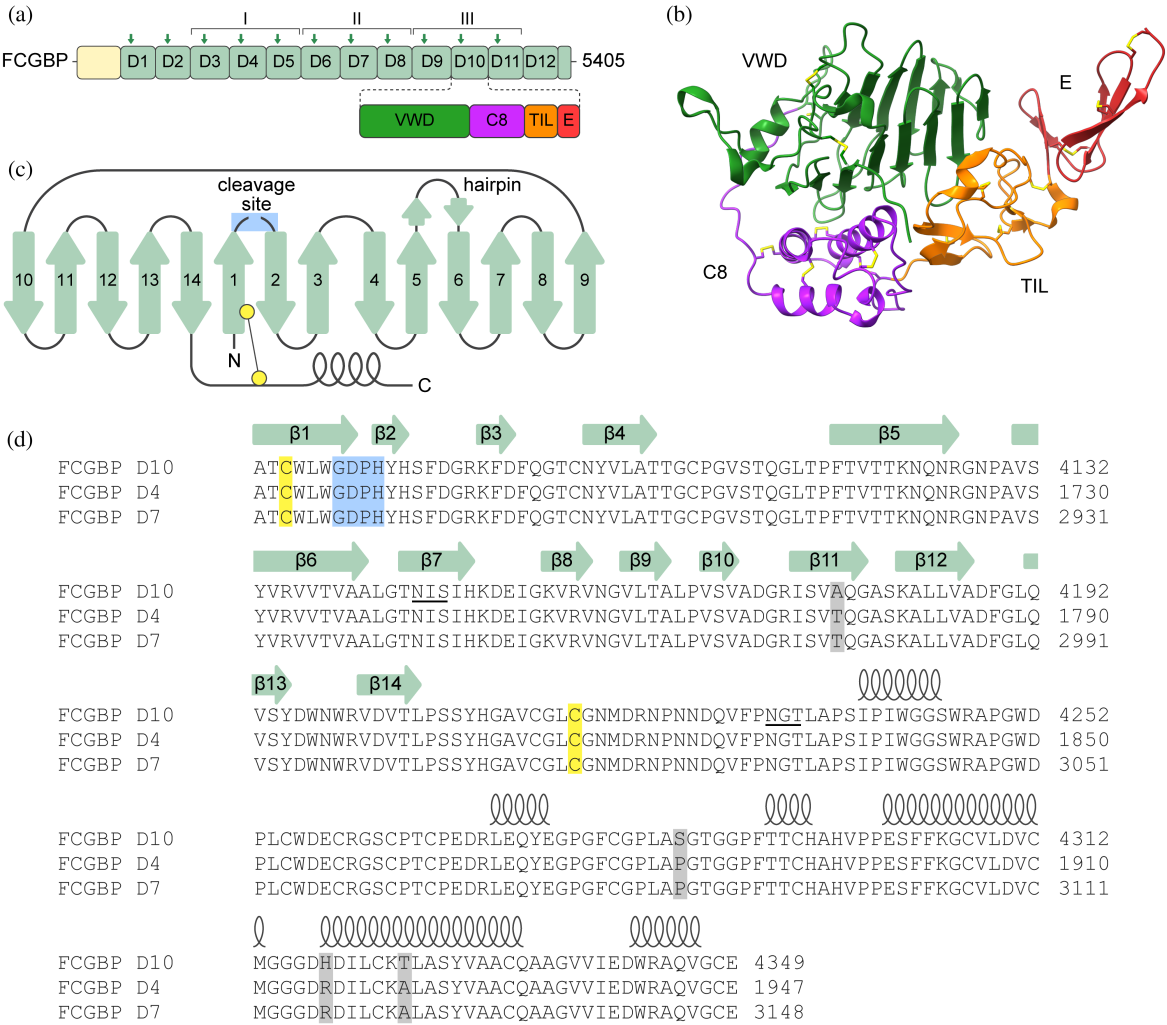
FCGBP D assemblies and sites of GDPH motifs. (a) D assemblies (light green) within the primary structures of FCGBP are numbered from amino to carboxy terminus. The length in amino acids of the human protein is given to the right. Positions of GDPH motifs are indicated by arrows. Roman numerals indicate the three repeats of three domains that are almost identical in sequence. Each D assembly comprises multiple domains, which are illustrated explicitly for FCGBP D10, the assembly studied structurally in this work. (b) Structure of the MUC2 D1 assembly, containing VWD, C8, TIL, and E domains (PDB 7PRL). Disulfides are shown as sticks with yellow sulfurs. (c) Topology diagram of the FCGBP D10 VWD domain indicating the site of cleavage between β‐strands β1 and β2 and an extended hairpin between β‐strands β5 and β6. The disulfide linking β‐strand β1 to the rest of the domain after GDPH cleavage is indicated by yellow balls. (d) Amino acid sequences of the VWD and C8 domains of D assemblies D10, D4, and D7. The D4 and D7 sequences were taken from UniProt Q9Y6R7; the D10 sequence was the version used in this study, as described in the section “Materials and Methods.” Secondary structure elements are indicated above the sequences, based on the D10 crystal structure. β‐strands are numbered as in panel C. Cysteines participating in the disulfide linking β‐strand β1 to the rest of the domain are highlighted in yellow. N‐linked glycosylation motifs in D10 are underlined. The GDPH motif is highlighted in blue, and amino acid positions that differ between D10 and the other two D assemblies are highlighted in gray.

“D assemblies” are arrangements of four domains that appear in multiple copies in gel‐forming mucins, which are the major glycoprotein constituents of mucus, and in the hemostasis protein von Willebrand factor (VWF). The four D assembly domains are named the von Willebrand factor type D (VWD), C8, TIL, and E domains (Figure 1a) (Nilsson et al., 2014). VWF and mucin structures have been determined using x‐ray crystallography and cryo‐electron microscopy (cryo‐EM), revealing a compact arrangement of the VWD, C8, and TIL domains (Figure 1b) (Anderson et al., 2022; Dong et al., 2019; Javitt et al., 2019, 2020, 2022; Reznik et al., 2022). The E domain projects out of the globular VWD/C8/TIL structure and appears to provide flexibility to the junctions between tandem D assemblies in VWF and mucins (Reznik et al., 2022). Such interdomain flexibility is likely important for conformational changes required during the bioassembly and function of mucins and VWF. Specifically, these proteins undergo non‐covalent supramolecular assembly intracellularly to template the formation of intermolecular disulfide bonds, and then, upon secretion, the non‐covalent complexes disassemble to yield long, linear, disulfide‐linked polymers (Javitt et al., 2020; Springer, 2014). Progress has been made in elucidating the mechanism by which tandem D assemblies contribute to disulfide‐mediated polymerization (Anderson et al., 2022; Javitt et al., 2020), but D assemblies also function in other contexts.

Another D‐assembly protein, expressed together with mucins in mucosal tissues, is the Fc IgG binding protein (FCGBP) (Pelaseyed et al., 2014). Human FCGBP contains 12 D assemblies, followed by an isolated VWD domain at the carboxy terminus (Figure 1a). Unlike mucins and VWF, FCGBP does not form disulfide‐bonded polymers (Ehrencrona et al., 2021). FCGBP has been reported to bind the Fc portion of antibodies (Harada et al., 1997) and factors involved in innate immunity (Houben et al., 2019), and it has been suggested to help clear mucosal surfaces of trapped pathogens (Kobayashi et al., 2021). However, the mechanism of FCGBP‐mediated clearance, its binding sites for other proteins, and the purpose of its many D assemblies are still unknown.

A notable feature of FCGBP is the presence of a glycine‐aspartate‐proline‐histidine (GDPH) motif in 11 of its 13 D assemblies (Figure 1a, c). One of the four D assemblies in each of the secreted mucins MUC5AC and MUC2 also contains a GDPH motif. A partial D assembly (VWD, C8, and TIL domains) with a GDPH motif is found in BMPER, a ligand for bone morphogenic protein (BMP) signaling (Lockhart‐Cairns et al., 2019). GDPH motifs have been shown to undergo auto‐catalytic cleavage between the Asp and Pro (Ehrencrona et al., 2021; Patrick & Egland, 2019), two amino acid residues known to be connected by a labile peptide bond (Piszkiewicz et al., 1970). Selective cleavage at Asp‐Pro bonds in polypeptides can be induced in vitro by low pH and high temperature (Landon, 1977), typically outside physiological ranges. The GDPH motif in VWD domains, however, is cleaved in vivo at the mildly acidic pH of the Golgi apparatus or secretory granules (Lidell et al., 2003), or even at the approximately neutral pH of the endoplasmic reticulum (ER) (Ehrencrona et al., 2021; Lidell & Hansson, 2006) or the junction between the ER and the Golgi (Komatsu et al., 2002), and at body temperature.

VWD domains containing GDPH motifs outside the context of D assemblies (i.e., without accompanying C8, TIL, and E domains) have been observed in additional proteins. For example, the transmembrane mucin MUC4 is cleaved into two subunits at a GDPH motif in a VWD domain (Chaturvedi et al., 2008). A cleaved GDPH motif has been studied structurally in the Repulsive Guidance Molecule B (RGMB), a ligand for the cell‐surface receptor Neogenin, which is involved in cell adhesion in diverse developmental processes. The structure of the RGMB VWD domain was determined in complexes with Neogenin (Bell et al., 2013) and with Neogenin and Netrin‐1 (Robinson et al., 2021). The RGMB VWD domain interacts with Neogenin via the outer face of the β‐sheet not involving the GDPH motif (PDB 4BQ6). The interaction with Netrin‐1 is made using the RGMB β‐sheet containing the GDPH (PDB 7NE0), but the motif does not directly participate in intermolecular interactions, and the Asp is solvent‐exposed. Nevertheless, mutations within and nearby the GDPH motif in the RGMB homolog RGMC are associated with the disease juvenile hemochromatosis and result in decreased secretion from cultured cells, indicating the importance of this region for protein stability and physiological function (Bell et al., 2013).

Understanding the structural and energetic consequences of GDPH cleavage will help guide research into the benefits afforded to proteins containing cleavable VWD domains. It will also lay the groundwork for engineering autocatalytic cleavage sites that may be used for regulatory or other purposes in designed proteins. In this study, we used a representative FCGBP D assembly to determine how GDPH cleavage affects VWD stability and structure, as well as to explore the chemical mechanism of cleavage. This FCGBP D assembly was analyzed biochemically and using x‐ray crystallography and molecular dynamics (MD) simulations, revealing intriguing differences compared to other, uncleaved D assemblies with known structure.

## 2 RESULTS

### 2.1 FCGBP VWD crystallization and structure solution

Human FCGBP has three copies of a set of three adjacent D assemblies (Roman numerals in Figure 1a). Considering natural variation in the human population, the FCGBP segment studied in this work is at least 99% identical in amino acid sequence to D4, D7, and D10 and thus can be considered representative of all three of these D assemblies (Figure 1d). For simplicity, we will refer to it as D10 (see section “Materials and Methods”). We produced and purified a version of D10 containing the VWD, C8, and TIL domains (residues 4073–4409), expected to constitute the globular part of the assembly (Figure 1b). Crystals of this FCGBP segment were readily grown, but most of them diffracted poorly. A crystal that provided diffraction data to 2.5 Å resolution was found only after intensive screening. Attempts to use molecular replacement (MR) to provide phase information were successful only when the VWD and C8 domains were used as separate search models. The reason became clear upon inspection of the MR solution, which revealed a different relative orientation of these two domains than seen previously in all other examples of D assemblies with experimentally determined structures (PDB codes 6N29, 6RBF, 6TM2, 7PP6, 7ZWH, 8D3C, and 7QCU), none of which have a cleaved GDPH motif. The region following the C8 domain was not evident in the electron density map calculated using the MR solution, and use of TIL domains as MR search models did not yield a solution for its position, indicating that it is flexibly tethered to the other two domains.

Based on these initial findings, a truncated variant containing only the VWD and C8 domains, spanning amino acid residues 4073–4349, was prepared. This variant crystallized in the same space group (P2_1_2_1_2_1_) with similar unit cell dimensions (43.72 × 54.32 × 129.11 Å) as the longer version (44.94 × 54.23 × 126.75 Å) but provided improved diffraction data, to 2.2 Å resolution. A structure model was built and refined using these data (Table 1).

**TABLE 1:** Data collection and refinement statistics.

*Data collection*	
Wavelength (Å)	1.34
Resolution range	19.49–2.20 (2.28–2.20)
Space group	P 2_1_ 2_1_ 2_1_
Unit cell (Å; °)	43.72 54.32 129.11; 90 90 90
Unique reflections	16,209 (1567)
Multiplicity	6.5 (3.9)
Completeness	99.6 (99.2)
Mean I/sigma (I)	10.9 (2.3)
R‐merge	0.165 (0.897)
R‐pim	0.0670 (0.509)
CC_1/2_	0.988 (0.599)
Wilson B factor (Å^2^)	28.6
*Refinement*	
Reflections used in refinement	16,192 (1559)
Reflections use for R‐free	1133 (109)
R‐work	0.197 (0.344)
R‐free	0.256 (0.431)
Number of non‐hydrogen atoms	2263
Macromolecules	2105
Ligands	35
Solvent	123
Protein residues	279
RMS bonds (Å)	0.009
RMS angles (°)	0.93
Ramachandran favored/allowed/outliers (%)	96.36/3.64/0
Rotamer outliers (%)	1.33
Clashscore	6.72
Average B‐factor	35.0

### 2.2 FCGBP D10 shows an unprecedented arrangement of the VWD and C8 domains

**FIGURE 2:**
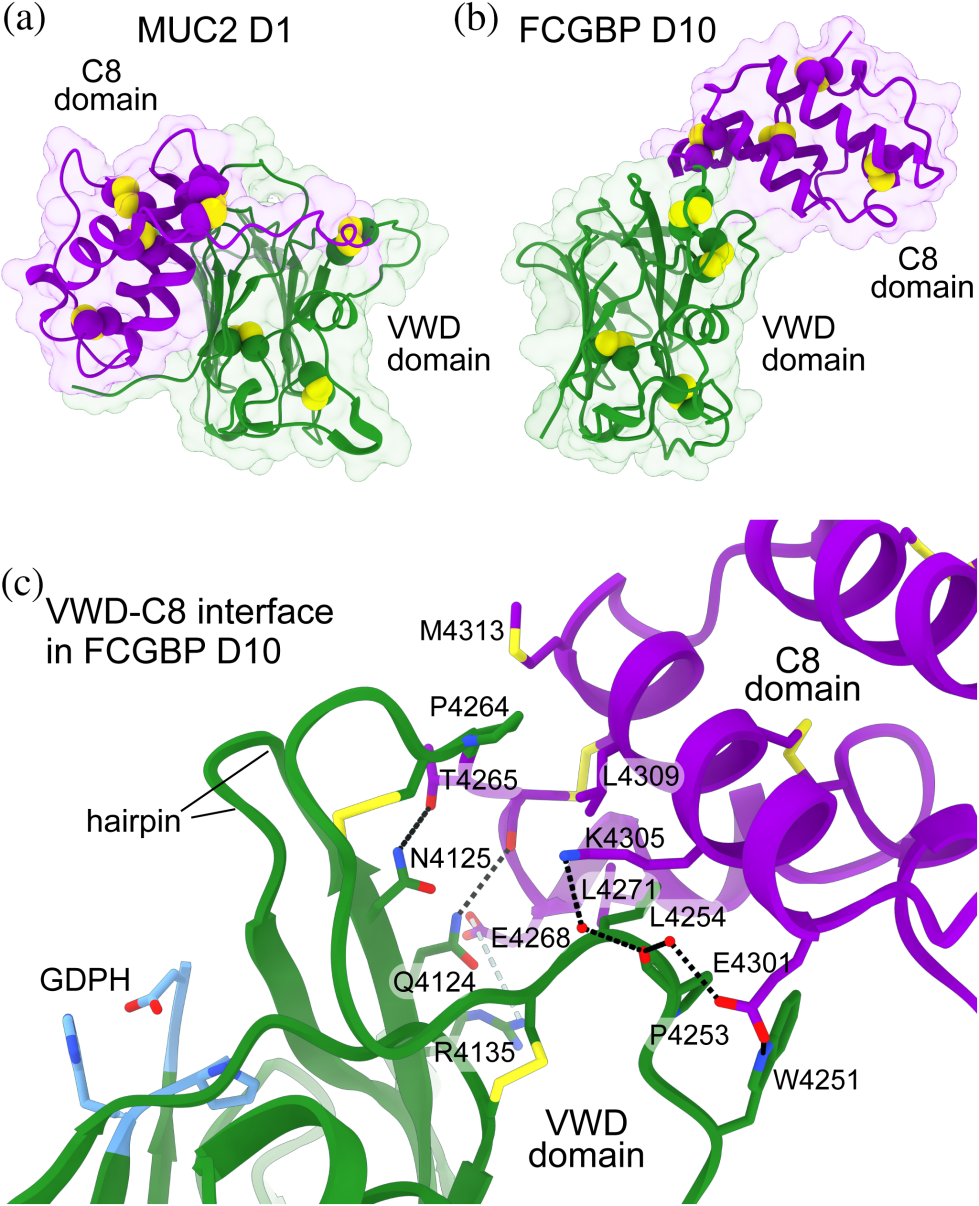
VWD‐C8 domain orientations differ between FCGBP D10 and other D assemblies. The VWD domains are green, and the C8 domains are purple. Disulfides are shown as spheres or sticks with yellow sulfur atoms. (a) Structure of the VWD and C8 domains of the MUC2 D1 assembly, extracted from PDB 7PRL. (b) Structure of the VWD and C8 domains of FCGBP D10. (c) Interactions between the VWD and C8 domains of FCGBP D10. Putative hydrogen bonds are shown as black dashed lines. A potential salt bridge is shown as a gray dashed line. Hydrophobic side chains in the interface are indicated. The GDPH motif is colored light blue.

The most striking feature of the FCGBP D10 segment structure was the relative orientation of the VWD and C8 domains. In other D assembly structures (Anderson et al., 2022; Dong et al., 2019; Javitt et al., 2019, 2020, 2022; Reznik et al., 2022), the C8 domain packs against the outside of one of the β‐sheets in the VWD domain β‐sandwich (Figure 2a). Specifically, two helices in the C8 domain interact with β‐strands β1, β2, and β3, and in some cases β‐strand β14, in the VWD domain, using mainly polar and some hydrophobic interactions. The TIL domain, in turn, interacts with the β‐strands β10–β14 (not shown). In FCGBP D10, the C8 domain was not packed against the side of the VWD β‐sandwich but instead was perched atop the sandwich (Figure 2b), interacting with an S‐shaped loop that in turn packs against the extended hairpin between β‐strands β5 and β6 (Figure 2c).

A comparison of the FCGBP D10 segment structure with models predicted by AlphaFold2 (Jumper et al., 2021) revealed good agreement for the individual domains but poor agreement in their relative orientation. The experimental structure and top‐ranking predicted model had all‐atom root mean square deviation (RMSD) values of 1.12 Å for the isolated C8 domain and 1.07 Å for the VWD region spanning H4084 to N4123 and V4134 to C4255, which excludes the hairpin loop between β‐strands β5 and β6 because its conformation is affected by interaction with C8. Despite the agreement within domains, four of the five top‐ranking AlphaFold2 models positioned the C8 domain against one side of the β‐sandwich as observed previously in D assembly structures (Anderson et al., 2022; Dong et al., 2019; Javitt et al., 2019, 2020, 2022; Reznik et al., 2022). Interestingly, in the prediction ranked third, the C8 domain was dissociated from the outside of the β‐sandwich and instead interacted with the VWD loop, analogous to the crystal structure. However, the relative orientation of the two domains deviated by more than 40° from the experimental structure (Figure S1).

### 2.3 Geometry of the cleaved GDPH motif

**FIGURE 3:**
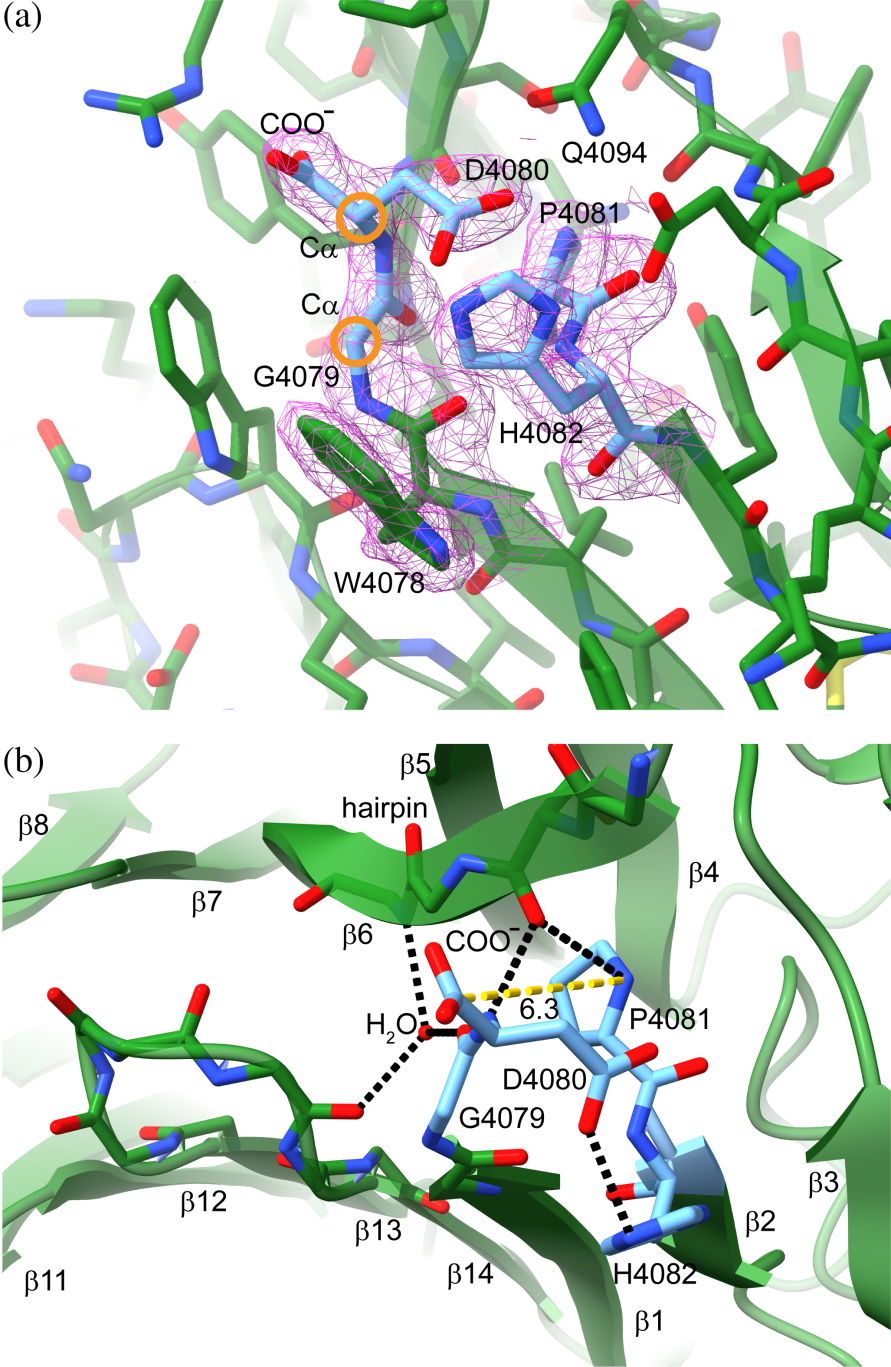
Geometry and interactions of the cleaved GDPH motif. (a) 2Fo‐Fc electron density is shown in magenta around the GDPH motif (1.2 σ). Orange circles indicate successive Cα atoms on the same side of the peptide bond, that is, in *cis*. COO^−^ indicates the terminal carboxylate arising from peptide bond cleavage. (b) Selected hydrogen bonding interactions (black dashed lines) made in the vicinity of the cleaved peptide bond. The yellow dashed line shows the distance in Ångstrom between the atoms that had been linked in a bond prior to cleavage. For clarity, side chains are not displayed, except in the GDPH motif.

A cleaved GDPH motif was previously observed in RGMB, but the aspartate was modeled in a variety of configurations in the structures of RGMB‐containing complexes (Bell et al., 2013; Robinson et al., 2021). Moreover, the second terminal oxygen on the aspartate was not included in the atomic coordinates. To ensure the chemical accuracy of the FCGBP D10 model, we carefully inspected the electron density maps in this region and included all atoms (Figure 3a). One notable feature of the cleaved motif is that the peptide bond before the aspartate (the Gly‐Asp bond) is unambiguously in the *cis* configuration. The *cis* peptide enables the Asp to make backbone hydrogen bonds to both the hairpin with which C8 interacts (Figure 2c) and, via a water molecule, to the loop between β‐strands β13 and β14 in the fold (Figure 1c). Aside from this unusual non‐Pro *cis* peptide, the are no geometrical outliers around the cleavage site. Specifically, the Gly and His are in favorable regions of Ramachandran space, and the side chains of the Asp, Pro, and His are all in favored rotamers, as analyzed using Molprobity (Williams et al., 2018). Another notable feature of the cleavage site in the D10 structure is that the backbone carboxylate carbon of the aspartate and the proline nitrogen are 6.3 Å apart, as opposed to about 1.3 Å in a peptide bond. Due apparently to a change in the Φ backbone dihedral angle of the Asp, the side chain carboxylate is closer than the backbone carboxylate to the Pro after cleavage (Figure 3a). In this orientation, the Asp side chain is in position to hydrogen bond with the side chain of the GDPH His. Overall, many hydrogen bonds and salt bridges are made by the chain termini that result from GDPH cleavage (Figure 3b).

### 2.4 Mutations within and outside the GDPH motif prevent cleavage

**FIGURE 4:**
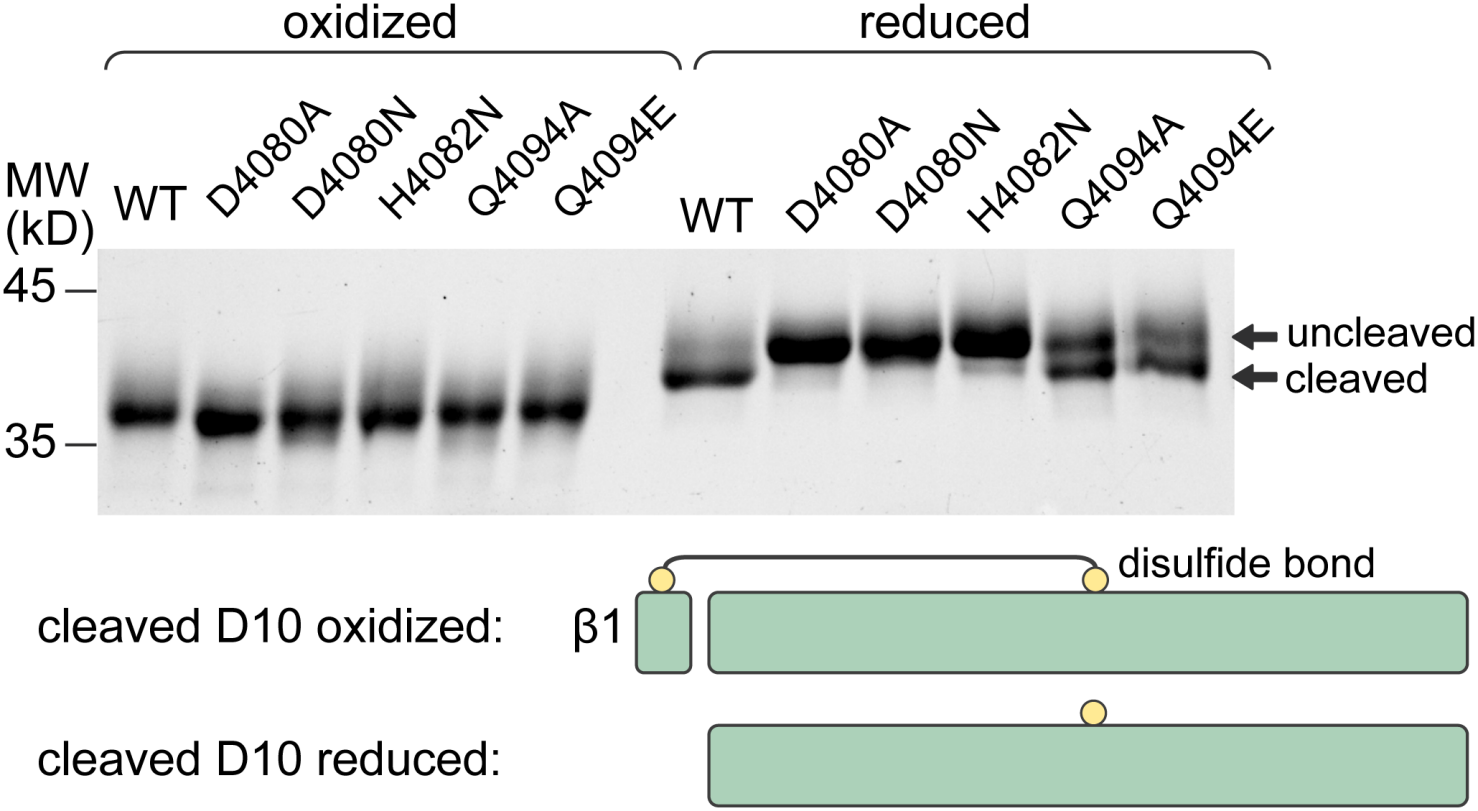
Mutations affect GDPH cleavage in FCGBP D10. The indicated mutants were produced, purified, and applied to a denaturing polyacrylamide gel (SDS‐PAGE). The lanes labeled “oxidized” were without further treatment; samples in the “reduced” lanes were treated with 2‐mercaptoethanol. Oxidized proteins migrate faster than reduced proteins due to the compactness afforded by intramolecular disulfide bonds. The oxidized wild‐type protein may migrate slightly more slowly than the uncleaved variants, for example, D4080A, due to the additional degrees of conformational freedom afforded by peptide‐bond cleavage, which would be expected to increase the average molecular size of the denatured protein. Cleaved and uncleaved versions can be distinguished readily in the reduced lanes due to the mass shift upon loss of the peptide containing the first β‐strand in the β‐sheet, as illustrated below the gel image.

To explore the requirements for spontaneous Asp‐Pro cleavage in the context of VWD domains, as well as to generate uncleaved variants, we mutated amino acids within and neighboring the GDPH motif of FCGBP D10. A two‐domain (VWD‐C8) version of the protein with a His_6_ tag and tobacco etch virus (TEV) protease cleavage site appended to the amino terminus was used for this experiment to increase the size of the fragment lost upon GDPH cleavage and reduction of disulfides. Mutation of the Asp in the GDPH motif to either Ala (D4080A; GDPH→GAPH) or Asn (D4080N; GDPH→GNPH) prevented cleavage (Figure 4), emphasizing the role of the Asp side chain in initiating cleavage. Similarly, mutation of the His in the GDPH motif to Asn (H4082N; GDPH→GDPN) inhibited cleavage, perhaps due to a role in polarizing the carbonyl that is the target of nucleophilic attack by the Asp side chain. Mutation of Gln Q4094, which is within hydrogen bonding distance of the D4080 side chain in the FCGBP D10 crystal structure, had a partial effect on cleavage, producing a mixed population of cleaved and uncleaved protein for both Q4094A and Q4094E.

**FIGURE 5:**
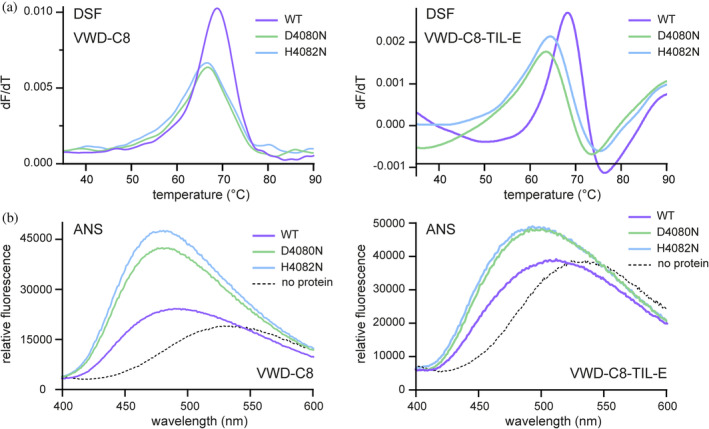
Uncleaved FCGBP D10 shows an altered thermal denaturation profile and has more apparent exposed non‐polar surface area than the cleaved wild‐type protein. Data are shown for both the two‐domain (VWD‐C8) and the four‐domain (VWD‐C8‐TIL‐E) versions of D10. (a) In DSF experiments, the thermal denaturation transition of wild‐type D10 was found to be sharper (higher *dF*/*dT*) than those of the indicated uncleaved mutants and to occur at a slightly higher temperature. (b) Uncleaved mutants showed greater fluorescence than wild‐type protein upon exposure to ANS, a dye that binds exposed non‐polar regions of proteins.

After identifying FCGBP D10 variants that were not cleaved, we sought to perform a structural comparison with the cleaved wild‐type version. However, extensive efforts to crystallize uncleaved mutants were unsuccessful. We also noticed that mutant D10 variants degraded more rapidly than the wild‐type protein during storage at 4°C (not shown). Differential scanning fluorimetry using intrinsic tryptophan fluorescence revealed a sharp thermal denaturation transition with a midpoint of about 69°C for the wild‐type VWD‐C8 protein, whereas the denaturation transitions of the uncleaved variants D4080N and H4082N occurred at about 67°C and were less sharp (Figure 5a). Addition of the TIL and E domains to obtain the four‐domain version (VWD‐C8‐TIL‐E) of FCGBP D10 did not increase the denaturation temperature; a sharp transition was seen for the wild‐type protein at about 68°C and for the mutants in this background at about 64°C. The fluorescence of 8‐anilino‐1‐naphthalene sulfonate (ANS), considered to be a probe for exposed non‐polar surface area in proteins (Cardamone & Puri, 1992), increased more when added to uncleaved mutants than to wild‐type D10 in both the VWD‐C8 and VWD‐C8‐TIL‐E backgrounds (Figure 5b), providing further evidence of differences in structure or dynamics between the cleaved and uncleaved versions. Together these data suggest that the uncleaved version of D10 has decreased cooperativity in its intramolecular interactions and non‐optimal burial of its hydrophobic residues.

### 2.5 GDPH cleavage increases resistance to protease

**FIGURE 6:**
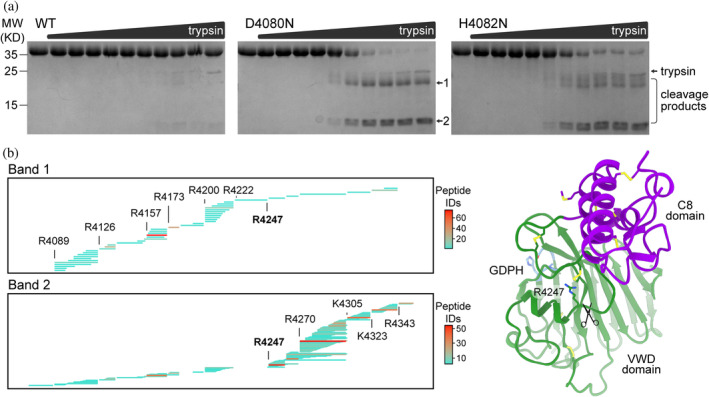
GDPH cleavage increases the resistance of FCGBP D10 VWD‐C8 to proteolytic cleavage. (a) Wild‐type (WT) FCGBP D10 and the indicated mutants were subjected to serial dilutions of trypsin and then analyzed by SDS‐PAGE under reducing conditions. (b) Mass spectrometry spectral counting of the D4080N cleavage products revealed that the upper band (Band 1) corresponds to the amino‐terminus (VWD domain) and the lower band (Band 2) to the carboxy‐terminus (C8 domain) of the D10 segment. The tryptic peptide between R4222 and R4247 is N‐glycosylated. (c) Structure of wild‐type D10 showing the position of R4247, the site of trypsin cleavage in the D4080N mutant. R4247 points into the S‐shaped loop and is about 13 Å from the GDPH cleavage site.

The altered thermal denaturation profile, increased ANS binding, and sensitivity of uncleaved FCGBP D10 mutants to spontaneous degradation imply that autocatalytic cleavage stabilizes the protein. To further explore this idea and identify vulnerable sites in the uncleaved version, we used limited proteolysis with trypsin. Both the four‐domain (VWD‐C8‐TIL‐E) and two‐domain (VWD‐C8) versions of wild‐type FCGBP D10 were essentially resistant to proteolysis at even the highest concentrations of trypsin used. In contrast, the D4080N and H4082N mutants were digested into fragments that could be separated by denaturing gel electrophoresis upon reduction of disulfide bonds (Figures 6a and S2). The two major fragments produced by trypsinization of the D4080N mutant were subjected to in‐gel trypsin digestion followed by tandem mass spectrometry spectral counting. The larger cleavage product was identified as the VWD domain and the smaller product as containing the C8 domain (Figure 6b), indicating that the individual domains remain protease‐resistant in the mutants and thus largely structured. R4247, immediately downstream of the last secondary structure element in the VWD domain (Figure 6c), was the most likely cleavage site, consistent with both the mass spectrometry and the sizes of the two bands on the gel. The fragments would be held together by the disulfide bond between C4097 and C4255. In the D10 structure, the peptide bond following R4247 is hydrogen bonded to β‐strand β4, the edge β‐strand of the β‐sheet opposite the GDPH proline, consistent with an effect of GDPH cleavage and local reorganization on the dynamics and exposure of the backbone near R4247.

### 2.6 Molecular dynamics provides insight into the mechanism of GDPH cleavage

As a reliable model of the uncleaved state of FCGBP D10 was not available by either x‐ray crystallography or structure prediction, we simulated this state by re‐forming the Asp‐Pro bond computationally. To avoid bias from the crystal structure, the scissile bond was re‐formed while restraining the peptide bond before the Asp (i.e., the Gly‐Asp bond) to be either *cis* or *trans*. Energy minimization was then performed on these two models. During minimization and subsequent MD trajectories, the Gly‐Asp bond remained either *cis* or *trans* without maintaining the restraints on these configurations.

**FIGURE 7:**
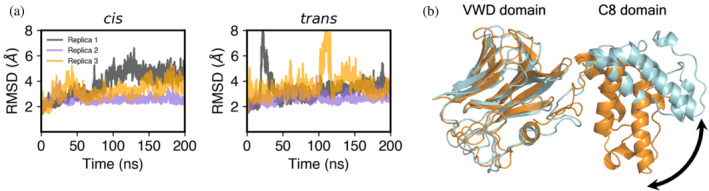
MD simulations of FCGBP D10 VWD‐C8 after computational formation of the Asp‐Pro bond in the GDPH motif with the Gly‐Asp bond in either the *cis* or the *trans* configuration. (a) Time evolution of heavy atom RMSD for three replicas of the *cis* and *trans* forms of the Gly‐Asp bond during 200 ns MD simulation. (b) Example of a hinge‐motion fluctuation in MD simulations. The cyan structure is close to the initial structure of FCGBP D10 in the *trans* form, and the orange structure shows a large change in the orientation of the C8 domain with respect to the VWD domain.

As a first validation of simulation stability, the heavy‐atom RMSDs of the two systems were calculated as a function of simulation time (Figure 7). Although several replicas showed significant structural changes in the relative positioning of the VWD and C8 domains, these changes were reversible, indicating that the simulations were stable. Interestingly, in the *tra ns* form, two of three replicas (replicas 1 and 3) showed large RMSD fluctuations (Figure 7a, right), reflecting reversible hinge motion that altered the relative orientation of the domains (Figure 7b). In the *cis* form, somewhat smaller fluctuations took place. Fluctuations within the C8 domain were observed to be greater than within the VWD domain (Figure S3).

**FIGURE 8:**
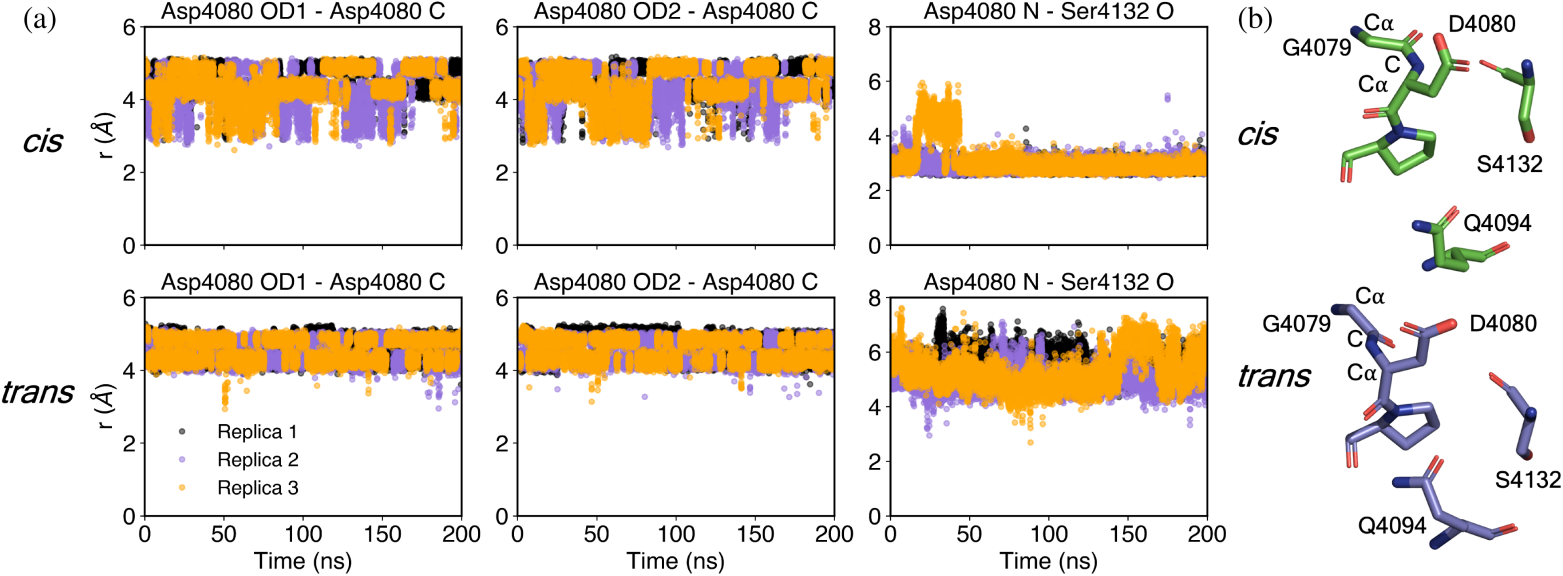
(a) Inter‐atomic distances between side chain atoms (OD1 and OD2) and the backbone carbonyl carbon atom (C) of D4080, and interatomic distances between the backbone nitrogen atom (N) of D4080 and the carbonyl oxygen (O) of S4132. (b) *cis* and *trans* forms of the peptide bond between G4079 and D4080.

A striking observation from the MD simulations was that the *cis* form of the Gly‐Asp bond, which enabled hydrogen bonding of the amino‐terminal cleavage product with other parts of the protein (Figure 3b), also promoted conformations conducive to the nucleophilic attack required to initiate cleavage. Specifically, the D4080 sidechain carboxylate oxygens in the *cis* form often fluctuated to within 3 Å of the D4080 backbone carbonyl carbon, an appropriate distance for initiating nucleophilic attack (Figure 8). In contrast, the *trans* form showed only extremely rare fluctuations closer than 4 Å. One of the features that appeared to stabilize the side chain of D4080 in the position close to the carbonyl carbon is an interaction of the D4080 backbone N–H group with the carbonyl of S4132 on β‐strand β6. This interaction is favored by the *cis* configuration of the Gly‐Asp bond, since in the *trans* form the N–H points away from S4132 (Figure 8). Q4094, mutation of which had a partial effect on cleavage, producing a mixed population of cleaved and uncleaved protein (Figure 4), was seen in position to activate the Asp carbonyl via a hydrogen bond. Additional interatomic distances in the vicinity of the autocatalytic cleavage site are displayed in Figures S4 and S5. Overall, it appears that structural features and local chemical groups that support the cleavage reaction also stabilize the cleaved product, except that after cleavage, the backbone and side chain carboxylate groups of D4080 essentially swap places and interactions (Figure 3).

### 2.7 Additional incidences of GDPH motifs

To explore the occurrence of potentially cleavable GDPH motifs that have been overlooked, we used Motif Search (https://www.genome.jp/tools/motif/MOTIF2.html), focusing on human proteins, to obtain a list of proteins containing the motif. We then inspected the AlphaFold2 predicted structures of cell surface and secreted proteins in the list obtained. Examples of identified proteins that may undergo autocatalytic cleavage are the inter‐alpha‐trypsin inhibitor heavy chains, secreted proteins that appear to have tumor‐suppressive properties and to be involved in extracellular matrix homeostasis (Huth et al., 2020; Rose et al., 2018). Despite substantial sequence divergence in the family, ITIH3, ITIH5, and ITIH6 all have a GDPH motif between the first two β‐strands in a predicted β‐sandwich domain. Moreover, the predicted fold of this domain shares secondary structure content and topology with VWD domains, but it was not annotated as a VWD domain in UniProt, Pfam, or Prosite.

## 3 DISCUSSION

Multiple copies of VWD domains are found in extracellular proteins required for endothelial integrity and the protection of mucosal tissues. VWD domains are also present in transmembrane and secreted proteins participating in fertilization and morphogenesis. It has long been appreciated that some VWD domains contain an autocatalytic cleavage site: a GDPH motif in the turn between the first two β‐strands of the domain (Bell et al., 2013). In some cleaved VWD domains, the first β‐strand remains covalently attached by a disulfide to the rest of the domain; in other instances, the disulfide is lacking, so cleavage could lead, in principle, to loss of the first β‐strand and fragmentation of the protein. In FCGBP, the subject of this study, all VWD domains contain cysteines at the appropriate positions in their primary structures to affix the first β‐strand covalently to the rest of the domain by a disulfide after cleavage. Indeed, human FCGBP was observed to migrate on denaturing gels as a high molecular weight protein that was reduced to small fragments by the addition of dithiothreitol (Ehrencrona et al., 2021).

To our knowledge, the functional purpose of GDPH cleavage has not yet been revealed for VWD domains that contain this motif, but the biochemical effects are beginning to be explored. We found that GDPH cleavage was stabilizing for the recombinant FCGBP fragment studied in this work. Specifically, cleavage increased the resistance of the protein to exogenous proteolysis and thermal denaturation, which may be valuable in the protease‐rich extracellular environments into which FCGBP is secreted, such as saliva and the intestinal lumen. Our data complement a previous observation that a disease‐causing mutation affecting GDPH cleavage in BMPER increased the susceptibility of BMPER to extracellular proteases (Lockhart‐Cairns et al., 2019). Though it was reported that GDPH cleavage in the VWD domain of the protein SUSD2 was required for it to aid in presentation of the protein Galectin‐1 on the cell surface, suggesting a functional role, the SUSD2 mutant designed for these experiments had a complete deletion of the GDPH motif and was retained in the endoplasmic reticulum (Patrick & Egland, 2019). Thus, it is likely that failure to traffic SUSD2 through the secretory pathway due to folding defects, rather than failure to cleave, was the primary cause of decreased Galectin‐1 presentation. A better understanding of the structural context of the GDPH cleavage site and the effect of mutations, such as presented here for FCGBP, lays the groundwork for future functional studies to uncover the reasons for the wide distribution and conservation of this motif in VWD domains.

In addition to the biological purpose of GDPH cleavage, the mechanism by which cleavage occurs under mild conditions is also intriguing. Mechanistic studies would benefit from a comparison of the high‐resolution structures of cleaved and uncleaved GDPH motifs. To date, however, no such pair of structures is available for any system containing this motif. Recently, the carboxy‐terminal region of the intestinal mucin MUC2, which has a VWD domain with a GDPH motif in the D4 assembly, was analyzed by cryo‐EM (PDB: 7QCU, 7QCN, and 7QCL) (Gallego et al., 2023). The MUC2 GDPH is cleaved in vivo (Herrmann et al., 1999) but was not cleaved in the host cells used to produce the protein fragment for structural studies (Gallego et al., 2023). The MUC2 carboxy‐terminal structure therefore provides a glimpse of an uncleaved state of a GDPH motif. Though the conclusions that can be drawn at 3.25 Å resolution are limited, all peptide bonds in the motif were modeled in the *trans* form. If indeed the Gly‐Asp peptide bond is primarily in *trans* in uncleaved MUC2, it is possible that the *cis* configuration is sampled transiently and drives cleavage when it occurs. The lack of cleavage at the MUC2 GDPH in the recombinant expression system could be a result of failure to sample, for unknown reasons, the *cis* form of the Gly‐Asp bond. Alternatively, a *cis* Gly‐Asp peptide bond might be assumed only after cleavage of GDPH motifs in VWD domains, despite the apparent contribution of the *cis* form to the cleavage mechanism (Figure 8). Another possibility is that the *cis* peptide between the Gly and Asp is a feature of FCGBP not shared by MUC2, and that MUC2 uses another method to promote nucleophilic attack. The MD simulations based on the FCGBP D10 VWD‐C8 structure clearly revealed that, at least for this protein and in silico, a *cis* configuration in the intact protein enables the close approach of the GDPH Asp side chain to the Asp backbone carbonyl carbon as required for nucleophilic attack and the initiation of chain cleavage.

In addition to the local structures around the Asp‐Pro bond in MUC2 and FCGBP, broader structural aspects of these two proteins can be compared. One notable difference between the cleaved FCGBP structure and the uncleaved MUC2 structures (PDB: 7QCU, 7QCN, and 7QCL) is the position of the extended loop between the VWD β‐strands β5 and β6, which is opposite the GDPH motif in the second β‐sheet of the β‐sandwich. In cleaved FCGBP D10, this loop forms a β‐hairpin that leans over the β‐sheet containing the GDPH motif (Figure 2c). One of the β‐strands of the hairpin is in position to form a backbone hydrogen bond from a carbonyl to the N‐H group of the aspartate in the GDPH motif, as well as to the new N‐H group of proline generated upon peptide cleavage (Figure 3b). In the uncleaved MUC2 structures, the analogous loop is modeled in three different conformations, indicating flexibility, and in none of these conformations does the loop backbone approach the uncleaved GDPH motif. One might speculate on the basis of MUC2 that the extended loop in FCGBP is more flexible in the uncleaved mutants, contributing to difficulties in crystallization. However, this loop was not a major target for trypsinization in the mutants (Figure 6b), despite the presence of an arginine (R4126) in the β‐hairpin, suggesting protection of the hairpin backbone in the uncleaved variants. As the FCGBP β‐hairpin interacts with the C8 domain, the structure of the β‐hairpin may be coupled to the domain orientation in FCGBP D assemblies.

The non‐canonical orientation of the C8 domain relative to the VWD domain in FCGBP D10 can be examined for potential implications for other D assemblies containing GDPH motifs. Though the observed orientation could in principle be a crystal‐packing artifact, or an artifact of the FCGBP fragments produced for study, two distinct fragments, VWD‐C8 and VWD‐C8‐TIL, showed the same arrangement. Moreover, biochemical analyses of the effect of GDPH cleavage gave qualitatively similar results for VWD‐C8 as for the complete four‐domain (VWD‐C8‐TIL‐E) version of the D10 assembly (Figures 5, 6, and S2), suggesting that the shorter fragments are representative of the longer version. The only other GDPH‐containing full D assembly that has been analyzed structurally, the D4 assembly of MUC2, showed a conventional relative orientation of the VWD and C8 domains, with the C8 domain packed against the VWD domain β‐sheet that contains the GDPH motif. The question arises whether the conventional orientation seen in this case is due to a lack of cleavage of the GDPH. If, upon cleavage, the MUC2 D4 assembly were to undergo a conformational change to resemble the FCGBP D10 VWD‐C8 structure, this change would likely separate the non‐covalently dimerized domains that form the stalk of MUC2 leading to the disulfide‐bonded CTCK domain dimer at the carboxy terminus. Therefore, a conformational change in MUC2 to the VWD‐C8 orientation seen for FCGBP would have a major effect on the organization of the MUC2 carboxy‐terminal region. While such a scenario cannot currently be ruled out, it may be more likely that, under distinct structural and functional constraints, FCGBP has simply diverged from mucins and displays a different three‐dimensional arrangement of domains.

The experiments reported here do show that the uncleaved version of the FCGBP D10 segment was resistant to crystallization, bound more ANS, showed a less cooperative thermal unfolding transition, and was more sensitive to exogenous proteases. The consistent picture presented by this range of probes demonstrates significant differences between the cleaved and uncleaved versions of the domain. However, these experiments do not distinguish between enhanced local fluctuations in the uncleaved state and a major, switch‐like conformational rearrangement upon cleavage. Interdomain flexibility in D assemblies was observed previously in the MUC2 D1 assembly when comparing a copper‐bound form of the isolated D1 assembly with the apo state of a larger MUC2 amino‐terminal segment (Javitt et al., 2020; Reznik et al., 2022). However, this flexibility involved TIL and E domain movements relative to a rigid VWD‐C8 pair. The analysis of FCGBP D10 segments described here reveals a potential divergence of the VWD‐C8 orientation compared to other homologous structures and flexible tethering of the TIL domain to the VWD‐C8 unit. The implications of these differences for the overall structural organization of FCGBP and for its function remain to be determined.

## 4 MATERIALS AND METHODS

### 4.1 Protein production and purification

FCGBP coding sequence was amplified from Human Lung QUICK‐Clone™ cDNA (TaKaRa) and inserted into the pcDNA3.1 vector using Restriction‐Free (RF) PCR methods. After DNA sequencing, the encoded protein sequence was assigned as D10 because a BLAST search (Altschul et al., 1990) retrieved D10 as the best match in the top hits (BAA19526.1 and XP_054178463.1). The D10 expression vectors contained the quiescin sulfhydryl oxidase (QSOX1) signal peptide at the amino terminus and a His_6_ tag at the carboxy terminus. The only exception to this design was a version with a His_6_ tag and a TEV cleavage site following the signal peptide used for the experiments in Section 2.4 to increase the size of the fragment that was lost upon GDPH cleavage. Mutations were introduced by RF PCR. Plasmids were propagated in and purified from the *E. coli* XL‐1 strain. The VWD‐C8 version of FCGBP D10 contained residues 4073–4349, the VWD‐C8‐TIL version contained residues 4073–4409, and the VWD‐C8‐TIL‐E version contained residues 4073–4471 (according to numbering for UniProt entry Q9Y6R7 but varying slightly in sequence).

FCGBP D10 proteins were produced in HEK293F cells maintained in FreeStyle 293 medium (Thermo Fisher Scientific). Plasmids were transiently transfected using PEI MAX reagent (Polysciences, Inc.) with a 1:3 ratio (w/w) of DNA to PEI at 1 million cells/mL. For protein produced for crystallization, 5 μM kifunensine (Cayman Chemical) was added during transfection to prevent the processing of high‐mannose Endoglycosidase H (EndoH)‐cleavable glycans. Six days after transfection, cultures were centrifuged for 15 min at 500*g* to pellet cells, and then culture medium was collected and centrifuged for another 15 min at 3200*g* to pellet any remaining particulate matter. The supernatant was filtered through a 0.45 μm filter, and the protein was purified using nickel‐nitrilotriacetic acid (Ni‐NTA) chromatography. Buffer was exchanged as described in the sections below.

### 4.2 Protein crystallization

To prepare protein crystallization stocks, buffer was exchanged to 10 mM Tris, pH 7.5, 20 mM NaCl, and the protein was concentrated to 6 mg/mL using a centrifugal concentrator. The protein was treated with EndoH (New England Biolabs) for 2 h at room temperature (RT) (500 U EndoH for 400 μL protein stock) to remove N‐linked glycans and used for crystallization without further purification. Crystals of the VWD‐C8 version of D10 were grown at 20°C by the hanging drop vapor diffusion method over a well solution containing 50 mM Tris, pH 8.0, 22% w/v PEG 8000, 20% v/v glycerol. Diffraction data were collected at 100 K on an in‐house Rigaku liquid‐metal‐jet (LMJ) x‐ray Synergy System with HyPix Arc 150° detector.

### 4.3 Mutations that affect Asp‐Pro cleavage

Mutant proteins were produced and purified using the methods described in Section 4.1. Buffer was exchanged to phosphate buffered saline (PBS). The proteins (1.5 μg per lane) were analyzed on a 13.5% denaturing polyacrylamide gel. Reduced samples contained 1.4 M 2‐mercaptoethanol (Merck).

### 4.4 Trypsin digestion assay

Wild‐type D10 and mutants at 15 μM in 0.5X PBS were incubated with a trypsin (Sigma Aldrich) dilution series ranging from 5.6 μM to 10.9 nM. Proteins were incubated with trypsin for 15 min at RT. Reactions were terminated by addition of an equal volume of sample buffer containing 2% sodium dodecyl sulfate, and samples were applied to denaturing 15% (for VWD‐C8) or 12% (for VWD‐C8‐TIL‐E) polyacrylamide gels under reducing conditions.

### 4.5 Mass spectrometry

Gel bands were destained by adding 150 μL of 25 mM ammonium bicarbonate in 50% acetonitrile to the tube and shaking at RT for 15 min. The process was repeated at least three times until no stain was visible in the destaining solution, which was discarded each time. The final destain solution was removed, and the gel was dried in a speed vac. The gel was reconstituted, and the protein was reduced by adding 100 μL 10 mM dithiothreitol in 25 mM ammonium bicarbonate and incubating for 1 h at 56°C. The reduction solution was removed and replaced with 100 μL 55 mM iodoacetamide. Samples were vortexed and spun down briefly. Alkylation proceeded for 45 min in the dark at RT. Solution was removed, and bands washed with 100 μL 25 mM ammonium bicarbonate solution, which was removed again. Bands were then dehydrated by adding 100 μL 25 mM ammonium bicarbonate in 50% acetonitrile solution and incubating twice for 5 min while vortexing, followed by speed vac. Bands were then rehydrated for digestion by adding 50 ng trypsin in 50 μL 25 mM ammonium bicarbonate on ice for 10 min. Samples were spun and incubated overnight at 37°C while shaking. The next day, the solution from each sample was collected into a clean tube. A 100 μL volume of 50% acetonitrile and 5% formic acid was then added to the gel bands, vortexed for 30 min, and collected to the same tube. The samples were then dried and reconstituted in 15 μL 3% acetonitrile and 0.1% formic acid for mass spectrometry.

Liquid chromatography mass spectrometry was performed on a nanoAcquity system (Waters, Milford, MA, USA) coupled onto a Q‐Exactive HF instrument (Thermo Scientific). Samples were loaded onto a Symmetry trap column (C18, 0.18 × 20 mm, 5 μm particles, Waters), and resolved on a T3 HSS analytical column (0.075 × 250 mm, 1.8 μm particles, Waters), running at 0.35 μL/min using a gradient of 4%–28% solvent B (99.9% acetonitrile, 0.1% formic acid) for 50 min. The sample was introduced into the instrument via nanoESI emitter (20 μm, Fossil Iontech, Madrid, Spain), mounted on a FlexIon source (Thermo Scientific). Data were acquired in Data‐Dependent Acquisition mode (DDA), using a Top10 method. MS1 resolution was set to 120,000 (@ 400 *m*/*z*), mass range of 375–1650 *m*/*z*, AGC of 3e6, and maximum injection time of 50 ms. MS was performed on ions with charge states of 2–7. MS2 resolution was set to 15,000 (@400 *m*/*z*), first mass to 100 *m*/*z*, HCD energy to 27 NCE, AGC of 1e5, max injection time to 60 ms, and dynamic exclusion set to 20 s.

The resulting data were searched using the Byonic search engine against FCGBP D10 D4080N. Data were searched using a non‐specific search, with 10 ppm for MS1 and 20 ppm for MS2. Modifications were set to fixed carbamidomethylation on C, variable deamidation on NQ, and oxidation on M. Peptide identifications were filtered for a log probability score of >2 post‐search.

### 4.6 Differential scanning fluorimetery

Protein stocks in 0.5X PBS (VWD‐C8) or 10 mM Tris, pH 7.5, 150 mM NaCl (VWD‐C8‐TIL‐E) were diluted (dilution factor of ×36 for VWD‐C8 and ×5 for VWD‐C8‐TIL‐E) to a final concentration of 5 μM in 100 mM Tris, pH 7.5, 150 mM NaCl. Measurements were done in duplicate using a Prometheus NT.48 (NanoTemper) instrument. Samples were heated from 25°C to 95°C at 1°C per min. Tryptophan and tyrosine fluorescence was monitored by recording the 350/330 nm emission ratio following 280 nm excitation, and the melting temperature was determined as the point of steepest slope.

### 4.7 ANS binding

Wild‐type and mutant proteins were diluted to a final concentration of 5 μM in 100 mM Tris, pH 7.5, 150 mM NaCl with 40 μM 8‐anilino‐1‐naphthalenesulfonic acid ammonium salt (Fluka). The excitation wavelength was set to 350 nm (with 9 nm bandwidth), and emission was monitored from 400 to 600 nm using a Tecan Infinite M200 Plate Reader. Measurements were made in triplicate. No protein was added to the buffer with ANS as a control.

### 4.8 Computational methods

Two sets of initial structures were constructed starting with the PDB ID 8QCI. The only difference in the set‐up protocols between these two proteins was the configuration of the peptide bond before D4080 (i.e., the Gly‐Asp bond), which was constrained to either the *cis* or the *trans* form. The systems set‐up commenced by adding hydrogen atoms using the HBUILD module of the CHARMM program (Brooks et al., 2009). His residues were modeled as neutral or protonated moieties with hydrogen positioned at either N_δ_ or Nε or both, depending on their hydrogen‐bonding pattern with surrounding amino acid residues or water molecules. The protein was solvated in a cubic box of 90 × 90 × 90 Å^3^ containing 123 water molecules from the crystal structure and 21,580 bulk solvent waters. The protein and ions were modeled using the CHARMM36 force field (Best et al., 2012). All water molecules were represented using the TIP3P water model (Jorgensen et al., 1983). The solvated system was neutralized by adding 14 Na^+^ and 10 Cl^−^ counter ions.

Following hydration, the uncleaved *cis* and *trans* forms were generated by energy minimization during which a bond between D4080 and P4081 was enforced via a standard force field bond term, and the *trans* form was generated by applying a dihedral restraint to the G4079‐D4080 peptide bond (Figure 8b). After initial preparation of the *cis* and *trans* forms in CHARMM, the final MD simulations were run using the NAMD simulation program (Phillips et al., 2005). Each system was initially minimized for 20,000 steps using the conjugate gradient method and then heated from 0 to 300 K in increments of 50 K in the NVT ensemble for a total of 1.2 ns. During energy minimization and heating of the *trans* form, the Gly‐Asp dihedral angle was constrained to 180°. Following heating, 20 ns of equilibration and 200 ns of production runs were performed at 1 atm with a time step of 1 fs in the NPT ensemble. All water molecules were modeled as rigid entities using the SHAKE algorithm (Allen & Tildesley, 1987). The imposed dihedral angle restraints were removed during the equilibration and production runs. For the electrostatic interactions, particle‐mesh Ewald was used with a grid spacing of 1 Å. Lennard‐Jones interactions were switched to zero at a distance of 12 Å. Three sets of independent simulations of 200 ns were run for both the *cis* and *trans* forms. All the data analyses were performed for the 200 ns of the NPT MD simulations.

## AUTHOR CONTRIBUTIONS

**Deborah Fass:** Conceptualization; funding acquisition; supervision; writing – original draft; writing – review and editing. **Noa Yeshaya:** Conceptualization; investigation; writing – review and editing. **Prashant Kumar Gupta:** Formal analysis; writing – review and editing. **Orly Dym:** Investigation; writing – review and editing. **David Morgenstern:** Investigation; writing – review and editing. **Dan Thomas Major:** Formal analysis; writing – review and editing; supervision.

## CONFLICT OF INTEREST STATEMENT

The authors declare no potential conflict of interest.

## Supporting information


**Data S1:** Models of uncleaved Asp‐Pro with cis and trans Gly‐Asp bonds in files D10_uncleaved_cis.pdb and D10_uncleaved_trans.pdb
Alphafold2 models in files D10_AF_rank1.pdb, D10_AF_rank2.pdb, D10_AF_rank3.pdb, D10_AF_rank4.pdb, D10_AF_rank5.pdb
Mass spectrometry peptides table in file D10_MS_peptides.xlsx

**Figure S1:** Comparison of AlphaFold2 models with the experimental FCGBP D10 VWD‐C8 segment. (A) The top AlphaFold model reproduces the conventional arrangement of the VWD and C8 domains seen in mucins and von Willebrand factor. This arrangement was also seen for AlphaFold2 models 2, 4, and 5. (B) AlphaFold2 model 3 places the C8 domain in a similar position relative to the VWD domain but does not reproduce the angle or specific interactions observed in the crystal structure. (C) Structure of FCGBP D10 VWD‐C8 fragment determined by x‐ray crystallography.
**Figure S2:** GDPH cleavage increases the resistance of four‐domain FCGBP D10 (VWD‐C8‐TIL‐E) to trypsin digestion. Wild‐type (WT) FCGBP D10 and the indicated mutants were subjected to serial dilutions of trypsin and then analyzed by SDS‐PAGE under reducing conditions
**Figure S3:** Comparison of root mean square fluctuation (RMSF) for C⍺ atoms of FCGBP D10 in the cis and trans forms from 200 ns of MD simulation. The C8 domain of the protein showed larger RMSF for both the cis and the trans forms, indicating that the C8 domain exhibits more internal mobility compared to the VWD domain.
**Figure S4:** Interatomic distances in the vicinity of Gly4079 and Asp4080 were calculated. Hsp denotes protonated His. The values and fluctuations in these distances were similar in the cis and trans forms for most of the interactions. However, a large change in the Trp4078 dihedral angle O‐C‐C⍺‐Cβ from −88° to −56° was observed in the trans form, which disrupted the interaction between Trp4078 and Asp4082 and resulted in large interatomic distances. This disruption was not observed for the cis form.
**Figure S5:** Trp4251 and Glu4301, at the interface of the VWD and C8 domains, showed abrupt changes in their interaction in the trans form at around 25 ns (replica 1) and 100 ns (replica 3). Fluctuations in this interaction were correlated with a large change in the orientation of the C8 domain relative to the VWD domain, as captured in the reversible RMSD changes (see main text, Figure 7). Hsp denotes protonated His

## Data Availability

Structure factors and coordinates for the FCGBP D10 assembly VWD‐C8 segment are available in the protein data bank as entry 8QCI.
