# Supplementary material for: VWD domain stabilization by autocatalytic Asp‐Pro cleavage
Source: Protein Sci. 2024 Feb 21;33(3):e4929. doi: 10.1002/pro.4929 (PMC10880436; doi:10.1002/pro.4929)
Supplement: Supplementary file 2 — Figure S1: Comparison of AlphaFold2 models with the experimental FCGBP D10 VWD‐C8 segment. (A) The top AlphaFold model reproduces the conventional arrangement of the VWD and C8 domains seen in mucins and von Willebrand factor. This arrangement was also seen for AlphaFold2 models 2, 4, and 5. (B) AlphaFold2 model 3 places the C8 domain in a similar position relative to the VWD domain but does not reproduce the angle or specific interactions observed in the crystal structure. (C) Structure of FCGBP D10 VWD‐C8 fragment determined by x‐ray crystallography. Figure S2: GDPH cleavage increases the resistance of four‐domain FCGBP D10 (VWD‐C8‐TIL‐E) to trypsin digestion. Wild‐type (WT) FCGBP D10 and the indicated mutants were subjected to serial dilutions of trypsin and then analyzed by SDS‐PAGE under reducing conditions Figure S3: Comparison of root mean square fluctuation (RMSF) for C⍺ atoms of FCGBP D10 in the cis and trans forms from 200 ns of MD simulation. The C8 domain of the protein showed larger RMSF for both the cis and the trans forms, indicating that the C8 domain exhibits more internal mobility compared to the VWD domain. Figure S4: Interatomic distances in the vicinity of Gly4079 and Asp4080 were calculated. Hsp denotes protonated His. The values and fluctuations in these distances were similar in the cis and trans forms for most of the interactions. However, a large change in the Trp4078 dihedral angle O‐C‐C⍺‐Cβ from −88° to −56° was observed in the trans form, which disrupted the interaction between Trp4078 and Asp4082 and resulted in large interatomic distances. This disruption was not observed for the cis form. Figure S5: Trp4251 and Glu4301, at the interface of the VWD and C8 domains, showed abrupt changes in their interaction in the trans form at around 25 ns (replica 1) and 100 ns (replica 3). Fluctuations in this interaction were correlated with a large change in the orientation of the C8 domain relative to the VWD domain, as captured in the reversible [file PRO-33-e4929-s002.pdf]

Supplementary Figures for

VWD Domain Stabilization by Autocatalytic Asp-Pro Cleavage

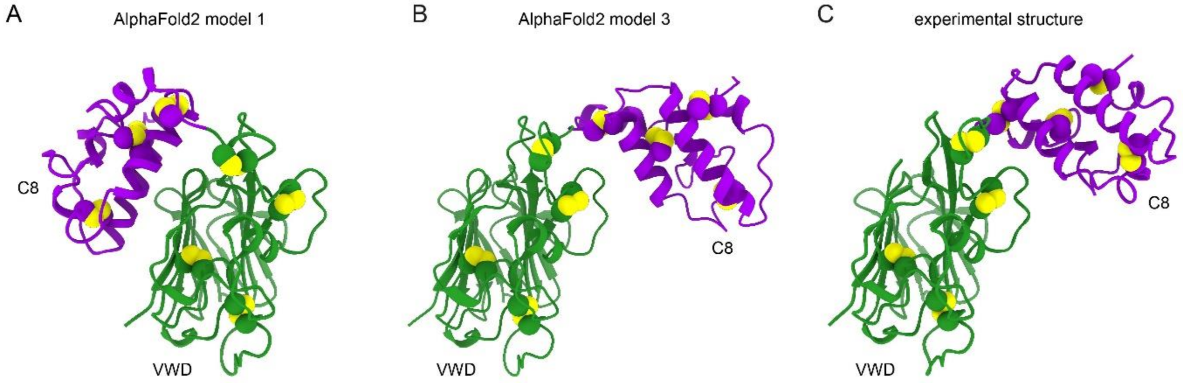

Figure S1: Comparison of the top AlphaFold2 models with the experimental FCGBP D10 VWD-C8 segment. **(A)** The top AlphaFold model reproduces the conventional arrangement of the VWD and C8 domains seen in mucins and von Willebrand factor. This arrangement was also seen for AlphaFold2 models 2, 4, and 5. **(B)** AlphaFold2 model 3 places the C8 domain in a similar position relative to the VWD domain but does not reproduce the angle or specific interactions observed in the crystal structure. **(C)** Structure of FCGBP D10 VWD-C8 fragment determined by X-ray crystallography.

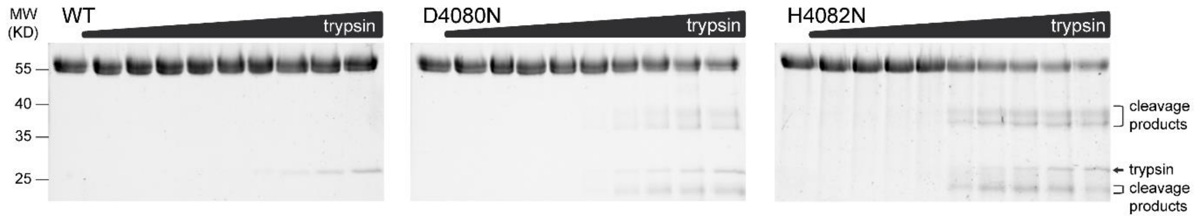

Figure S2: GDPH cleavage increases the resistance of FCGBP D10 including all four domains (VWD-C8-TIL-E) to proteolytic cleavage. Wild-type (WT) FCGBP D10 and the indicated mutants were subjected to serial dilutions of trypsin and then analyzed by SDS-PAGE under reducing conditions.

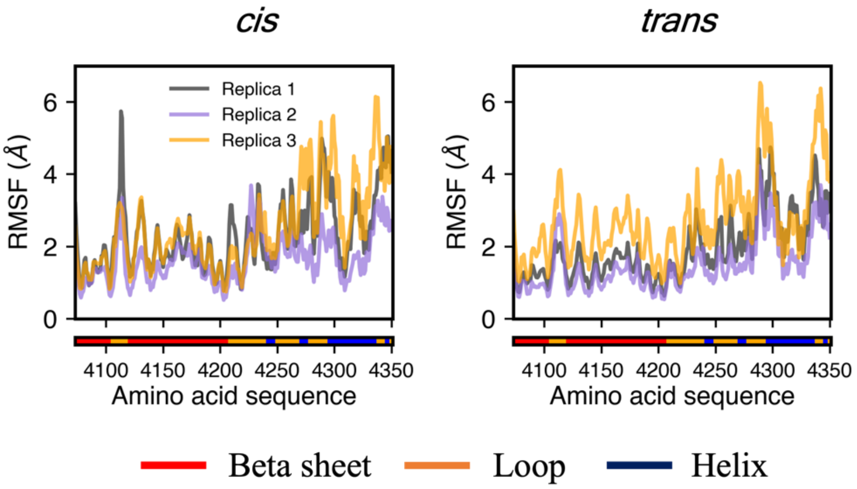

Figure S3: Comparison of RMSF for Cα atoms of FCGBP D10 in the *cis* and *trans* forms from 200 ns of MD simulation. The C8 domain of the protein showed larger RMSF for both the *cis* and the *trans* forms, indicating that the C8 domain exhibits more internal mobility compared to the VWD domain.

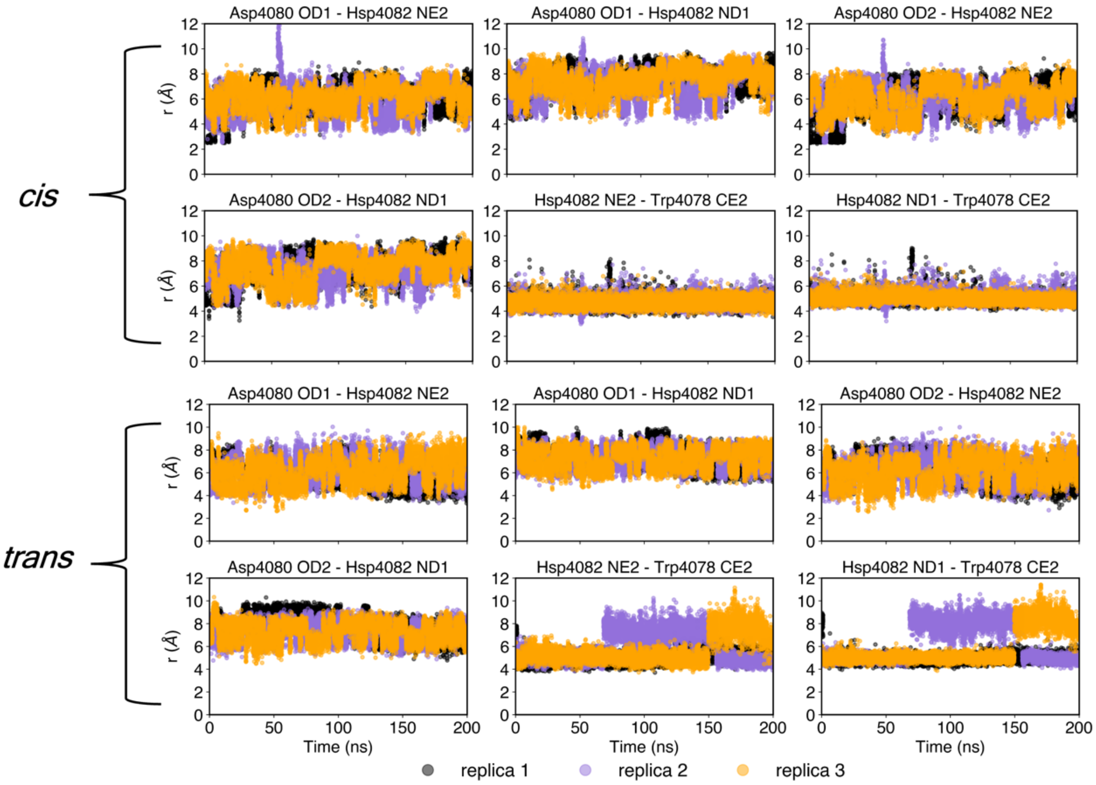

Figure S4: Interatomic distances in the vicinity of Gly4079 and Asp4080 were calculated. Hsp denotes protonated His. The values and fluctuations in these distances were similar in the *cis* and *trans* forms for most of the interactions. However, a large change in the Trp4078 dihedral angle O-C-C_α-C_β from -88° to -56° was observed in the *trans* form, which disrupted the interaction between Trp4078 and Asp4082 and resulted in large interatomic distances. This disruption was not observed for the *cis* form.

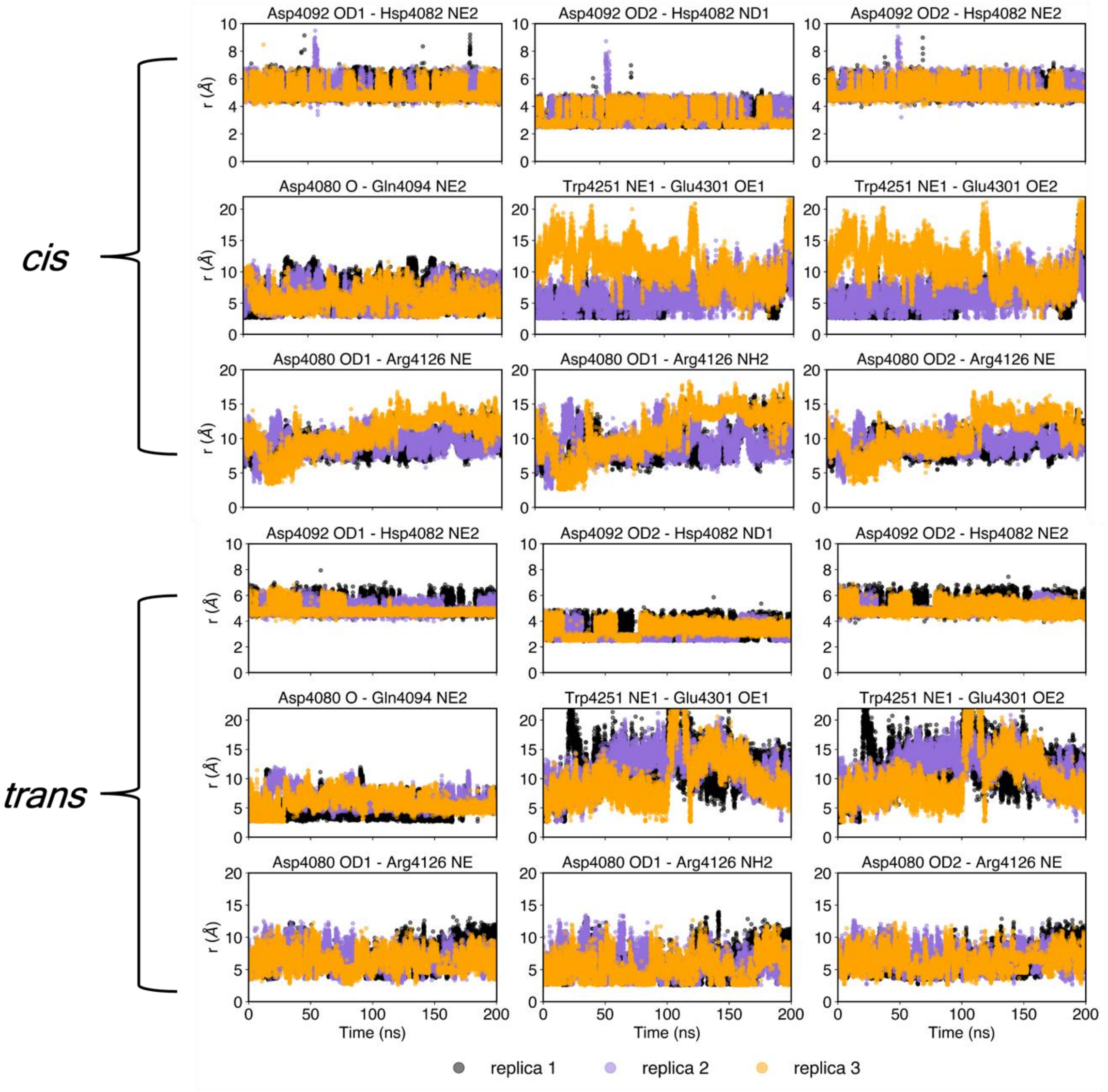

Figure S5: Trp4251 and Glu4301, at the interface of the VWD and C8 domains, showed abrupt changes in their interaction in the *trans* form at around 25 ns (replica 1) and 100 ns (replica 3). Fluctuations in this interaction were correlated with a large change in the orientation of the C8 domain relative to the VWD domain, as captured in the reversible RMSD changes (see main text, Figure 7). Hsp denotes protonated His.
